# Dissociable Learning Processes Underlie Human Pain Conditioning

**DOI:** 10.1016/j.cub.2015.10.066

**Published:** 2016-01-11

**Authors:** Suyi Zhang, Hiroaki Mano, Gowrishankar Ganesh, Trevor Robbins, Ben Seymour

**Affiliations:** 1 Center for Information and Neural Networks, National Institute for Information and Communications Technology, 1-4 Yamadaoka, Suita, Osaka 565-0871, Japan; 2 Computational and Biological Learning Laboratory, Department of Engineering, University of Cambridge, Trumpington Street, Cambridge CB2 1PZ, UK; 3 CNRS-AIST JRL (Joint Robotics Laboratory), UMI3218/CRT, 1-1-1 Umezono, Tsukuba, Ibaraki 305-8560, Japan; 4 Immunology Frontier Research Center, Osaka University, 3-1 Yamadaoka, Suita, Osaka 565-0871, Japan; 5 Behavioural and Clinical Neuroscience Institute, Department of Psychology, University of Cambridge, Downing Site, Cambridge CB2 3EB, UK

## Abstract

Pavlovian conditioning underlies many aspects of pain behavior, including fear and threat detection [1], escape and avoidance learning [2], and endogenous analgesia [3]. Although a central role for the amygdala is well established [4], both human and animal studies implicate other brain regions in learning, notably ventral striatum and cerebellum [5]. It remains unclear whether these regions make different contributions to a single aversive learning process or represent independent learning mechanisms that interact to generate the expression of pain-related behavior. We designed a human parallel aversive conditioning paradigm in which different Pavlovian visual cues probabilistically predicted thermal pain primarily to either the left or right arm and studied the acquisition of conditioned Pavlovian responses using combined physiological recordings and fMRI. Using computational modeling based on reinforcement learning theory, we found that conditioning involves two distinct types of learning process. First, a non-specific “preparatory” system learns aversive facial expressions and autonomic responses such as skin conductance. The associated learning signals—the learned associability and prediction error—were correlated with fMRI brain responses in amygdala-striatal regions, corresponding to the classic aversive (fear) learning circuit. Second, a specific lateralized system learns “consummatory” limb-withdrawal responses, detectable with electromyography of the arm to which pain is predicted. Its related learned associability was correlated with responses in ipsilateral cerebellar cortex, suggesting a novel computational role for the cerebellum in pain. In conclusion, our results show that the overall phenotype of conditioned pain behavior depends on two dissociable reinforcement learning circuits.

## Highlights

• Different brain learning systems are associated with different defensive responses
• Cerebellar responses correlate with “associability” for ipsilateral predicted pain
• The overall phenotype of conditioned pain is the sum of two part-independent processes

## Teaser

The classical “fear” response elicited by the brain in the presence of threat is critical for survival. Using pain as the threat, Zhang et al. show that autonomic and motor defensive reactions are associated with multiple learning systems. This means the overall phenotype of pain-based fear response is the sum of partially independent processes.

## Results

The brain is acutely tuned to detecting a variety of threats, especially pain, and elicits a set of appropriate responses as soon as potential harm is detected. This classic “fear” response is critical for survival, and the way in which clues in the environment are used to predict harm (Pavlovian conditioning) represents one of the most important and evolutionary conserved learning systems in animals. However, it is not clear whether the overall phenotype of the pain-based fear response represents a single process or the sum of partially independent processes.

**Figure 1:**
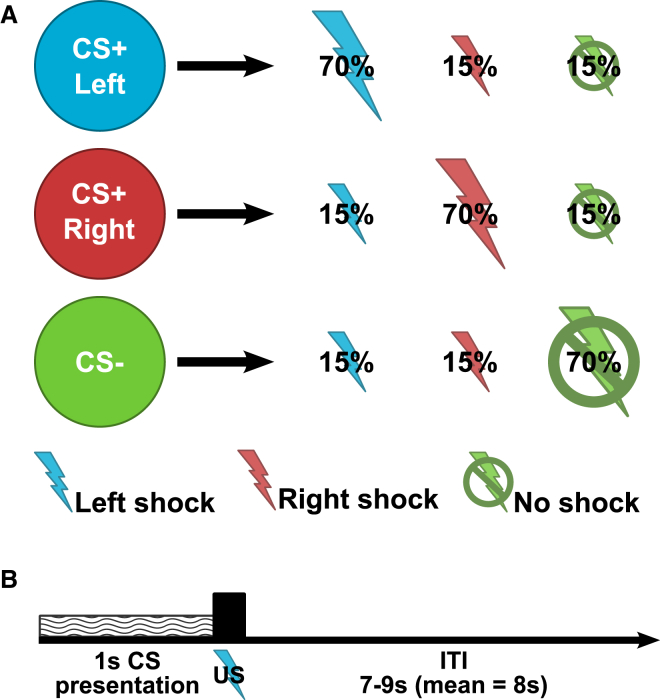
Experimental Design (A) Each trial involved one of three Pavlovian CS cues, each of which primarily predicted (70%) either left pain (blue symbol), right pain (red), or no pain (green) and infrequently predicted the other outcomes (15%). (B) On each trial, a 1-s CS cue was followed immediately by pain or no pain (US) in a delay conditioning procedure, followed by a variable 7- to 9-s inter-trial interval (ITI).

We acquired fMRI and simultaneous physiological responses in 15 healthy human subjects in a Pavlovian first-order delay conditioning experiment (Figure 1; Experimental Procedures). Visual cues differentially predicted frequent lateralized pain to either left or right arm or infrequent pain. A relatively short CS-US interval of 1 s was used to optimize detection of reflex-like conditioned muscle activities, similar to the design of eye-blink conditioning studies [6]. Ultra-brief painful heat stimuli at 55°C were used as unconditioned stimuli, delivered through two contact heat-evoked potential stimulators.

### Physiological Responses

**Figure 2:**
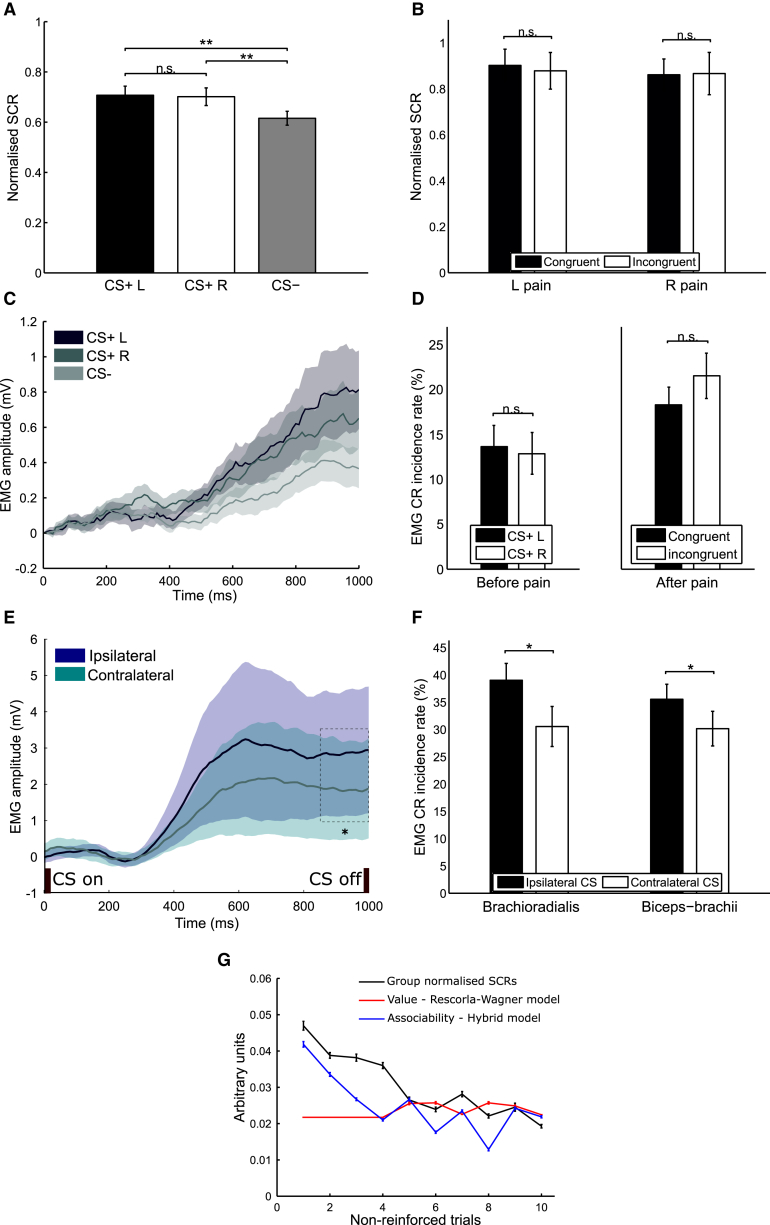
Behavioral Results (A) CS-evoked SCRs in “unreinforced” trials show significant differences between CS+ L/R and CS− (*T*_*L*_(41) = 2.78; *T*_*R*_(41) = 2.99; both p < 0.01), but not between CS+ L and CS+ R (*T*(41) = 0.14; p = 0.89). (B) SCRs for reinforced pain trials with congruent/incongruent predictions, separated into L/R pain groups, showing no significant differences. (C) Facial EMG traces during 1-s CS-US interval show CS+ L/R > CS− in amplitude (combined CS+ L/R versus CS− p < 0.05 in 500–1,000 ms), but not significant difference between CS+ L/R (all time points p > 0.1). (D) Average facial EMG conditioned response (CR) incidence shows no significant difference between CS+ L/R during 1-s CS-US interval before or during 1 s after pain delivery, between congruent/incongruent trials (both p > 0.5). (E) Time course of upper-limb EMG during 1-s CS-US interval averaged across L/R, with ipsilateral > contralateral response amplitude (p < 0.05 in 850–1,000 ms). (F) Average upper-limb EMG CR incidence in brachioradialis and biceps-brachii muscles, significantly greater for ipsilateral trials (both p < 0.05). (G) Trial-by-trial model fit of associability (blue) and value (red) to group-normalized SCRs (black) of non-reinforced trials in one session (first ten trials). Data are represented as mean ± SEM. ^∗^p < 0.05; ^∗∗^p < 0.01; n.s., not significant.

We recorded a number of different physiological responses to evaluate the acquisition of conditioned responses. Skin conductance responses (SCRs) did not distinguish the laterality of predicted or received pain, consistent with a preparatory response. Specifically, SCRs showed comparable conditioning to cues that predicted left (CS+ L) or right (CS+ R) arm pain, in comparison to control (CS−) (Figure 2A; data represented as mean ± SEM). SCRs to the pain itself were also comparable regardless of whether the pain was delivered to the predicted (congruent) or unpredicted (incongruent) side (Figure 2B). We could not identify any significant laterality differences in early or late learning periods during each session, from either normalized SCR magnitude or rise time to peak (Figures S3C and S3D).

Facial electromyography (EMG) also followed a preparatory pattern. The EMG was recorded from the corrugator muscle, a characteristic muscle of aversive expression, during a behavioral version of the task (Figure S1). The response during the 1-s CS-US interval averaged across trials showed a significant increase in 500- to 1,000-ms time window for both CS+ L and CS+ R trials compared to CS− trials (combined CS+ L/R versus CS− paired t test p < 0.05 in 500–1,000 ms), but not significant between CS+ L and R groups (p > 0.1 for all sample points; Figure 2C). Comparing pain-evoked responses for congruent and incongruent prediction trials during 1-s duration after painful US delivery revealed no statistically significant differences, consistent with a preparatory response (both p > 0.5; Figure 2D).

In contrast, EMG responses from each arm (recorded from brachioradialis and biceps-brachii, which are involved in upper limb withdrawal) showed lateralized “consummatory” patterns. We recorded activity in the 1-s CS-US interval and compared it to pre-CS baseline activity. We found that responses were significantly greater in the arm in which pain was predicted (ipsilateral) as opposed to the contralateral side (Figures 2E and 2F). Note that, because of the proximity of the stimulating thermode and the EMG electrodes, US responses (to look for congruency effects) are unavoidably too corrupted by electrical artifact for analysis.

### Imaging Results

Reinforcement learning theory proposes that acquisition of conditioned responses from trial-by-trial experience utilizes two key measures: a prediction error term that records the difference between pain expectations and outcomes [5] and an “associability” term that keeps track of the uncertainty of predictions [7, 8]. These two measures are then integrated to update CS values that provide the prediction for the next trial. Accordingly, the larger the prediction error, the greater the update in CS value. The associability term acts as the learning rate of value, with higher associability representing greater uncertainty and hence more rapid learning.

SCRs were of sufficient fidelity to permit trial-by-trial analysis using a computational statistical model fitting procedure. In agreement with previous reports [7, 8], we found it best described by a preparatory associability term, illustrated in Figure 2G.

We then used the estimated model parameters in a linear regression with brain responses recorded by concurrent fMRI to identify whether anatomically distinct learning signals related to preparatory and left/right consummatory learning signals could be dissociated. We used the computational parametric regressors for all learning signals (associability and prediction error for both preparatory and consummatory temporal difference models) in a single regression model. These values were generated using population free parameters with the best fitting model, the hybrid model, obtained from the behavioral data (SCRs) fitting procedure mentioned earlier.

**Figure 3:**
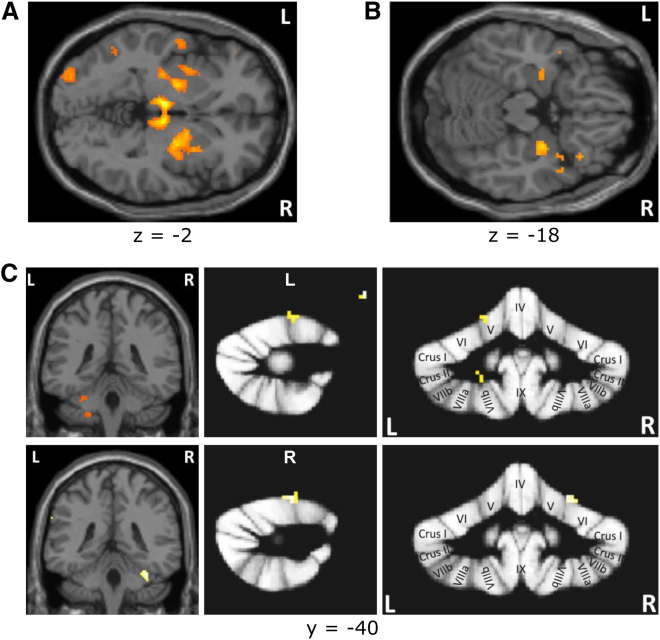
Statistical Parametric Maps (A) Preparatory prediction error in bilateral ventral putamen (p < 0.001 unc.). (B) Preparatory associabilities in bilateral amygdala (p < 0.01 unc.). (C) Ipsilateral activations to consummatory associabilities (p < 0.001 unc.; all p < 0.05 in small volume correction [SVC] using anatomically defined 8-mm-diameter spherical ROI masks built around hypothesized structure coordinates; see Table S1). ROI analysis of cerebellum using SUIT probabilistic atlas template shows (top) left anterior cerebellum activations in the border between lobule V and VI (SUIT space coordinates: [24, −52, −15]) and in lobule VIII ([−22, −50, −41]; p < 0.004 unc.) and (bottom) right anterior cerebellum activation in the border between lobule V and VI ([−18, −52, −13]; p < 0.001 unc.). unc., uncorrected threshold.

We found that bilateral ventral putamen and amygdala correlated with a preparatory temporal prediction error and associability signal, respectively (Figures 3A and 3B). In contrast, left and right consummatory associabilities correlated with ipsilateral cerebellar responses. Associability signal clusters were located symmetrically in lobule left V extending into left VI and spanning the border between lobules right V and right VI (Figure 3C). The peak coordinates of these cerebellar activations were in gray matter, as identified by the automated anatomical labeling (AAL) and spatially unbiased infratentorial template (SUIT) atlases. In addition, post hoc analyses of functional regions of interest (ROIs) support the hypothesized roles of structures identified by computational models. Beta estimates were extracted for each subject from the functional clusters of interest as they appear in given contrasts. They were averaged across subjects according to model or trial types without parametric modulation, where amygdala, putamen, and cerebellum showed differential responses to preparatory and consummatory model outputs (Figures S3G and S3H).

## Discussion

In summary, our results dissociate two distinct response-learning systems underlying human pain. An amygdala-striatal system learns preparatory responses, including autonomic responses and facial expression, and largely ignores information about the laterality of pain. In contrast, a cerebellar system learns specific consummatory limb withdrawal responses appropriate to the anatomical site of predicted pain.

The role of the amygdala in preparatory conditioning is well established. For instance, amygdalar lesions impair autonomic responses, freezing, potentiated startle, and active avoidance [1, 2]. Our data show that a preparatory associability signal drives activity at the level of the fMRI BOLD, consistent with previous studies in both humans and rodents [7, 8, 9, 10]. It is important to note, however, that aversive prediction errors have been identified at a neuronal level in rodents [11, 12]. Although there exist species and methodological differences in comparison to our study, it illustrates the differences in methodology between BOLD responses and neuronal physiological recordings. In particular, because the BOLD signal could be conveying the average signal of a potentially computationally heterogeneous group of neurons, some caution is needed against over-interpretation of the results. On the other hand, it is still unclear how some computational quantities might be encoded by distributed activity of a population of neurons.

Results from other studies also argue against any simplistic single model of amygdala function. For example, amygdala responses have been shown contralateral to the shock laterality in unilateral eye-blink conditioning [13] and to exhibit non-symmetrical activations in a range of fear paradigms [14], in contrast to the results here, which lacked laterality dissociation. Other factors such as motivational state [15] and sensitivity to inferred (“model-based”) cue-outcome contingency [16] have also been demonstrated. Therefore, whereas our computational model-based analysis showed that the expression of preparatory responses appears to be outcome blind, we certainly cannot exclude the possibility that neuronal processing within the amygdala may incorporate information about outcome identity, including laterality.

The involvement of the putamen in aversive conditioning was discovered much later than amygdala, and its function has been less clear. Because the putamen receives cortical somatotopic pain projections [17], it is possible that it might have carried a consummatory or sensory-specific error signal [18, 19]. However, the non-lateralized nature of the signal seen here instead provides good evidence to suggest that it is primarily part of a preparatory system.

Most significantly, the results provide a formal account of one of the roles of the cerebellum in pain. Previous research, including using human fMRI, has showed cerebellum responses to noxious stimuli; however, defining a specific role in pain processing has been difficult [20]. Stimulation of the cerebellum can alter nociceptive thresholds and reflexes in animals [21], suggesting it may engage in pain modulation along with various brainstem structures involved in the cerebrocerebellar loop [20, 22]. Evidence from human studies indicates cerebellum may be activated by other processes related to, but not exclusive to, pain sensory processing, for example, motor withdrawal [23], anticipation to pain [24], and negative emotions [25]. This has led to the proposal that the cerebellum may act as an integrator of various effector systems of pain such as sensorimotor integration, pain modulation, and affective processing [20].

Our results provide evidence of an uncertainty-sensitive associative learning process for ipsilateral conditioned motor responses. Anatomically, the major activation was localized in the anterior lobe, bordering lobule V and VI, which concurs with the sensorimotor area of previous functional topographic studies [26]. Conditioned postural limb activation during electrical shock conditioning is known to depend on an intact anterior and superior cerebellum [27]. Electrical shocks, however, also recruit ascending proprioceptive fibers that project to cerebellum and support motor learning. Here, our use of thermal pain stimulation—which should selectively activate a-delta and c-fibers afferents—provides evidence of a primary nociceptive-driven learning process.

This result suggests parallels with eye-blink conditioning, a prototypical consummatory response. Anatomically, both animal and human lesion experiments have identified an association between lobule V and VI with impairment or disruption of eye-blink conditioning [28, 29]. Computationally, cerebellar climbing fiber activity has been shown to represent prediction error magnitude [30], from which associability might be calculated. Previous eye-blink studies have suggested a distinction between preparatory and consummatory learning processes. Although both excitatory and inhibitory conditioning on one eye can transfer to the other [31], cues predicting unilateral air puff do not block acquisition of contralateral blink responses, but they do block autonomic responses [32]. This suggests preparatory and consummatory learning systems are distinct but interact.

Together, our data show that the expression of learned pain behavior is the sum of multiple, distinct neural processes. This has important implications for how we evaluate pain and its treatment, especially in animals where motor responses such as paw withdrawal and tail flick are the predominant outcome measures by which pain is inferred. Our data show that different emitted responses may correspond to different underlying neural subsystems of pain, which may help explain difficulties in translating animal-to-human results.

## Experimental Procedures

### Subjects and Experimental Design

Fifteen healthy human subjects participated in a Pavlovian first-order delay conditioning experiment (Figure 1; Supplemental Experimental Procedures). All subjects gave informed consent prior to participation, and the study was approved by the Ethics and Safety Committee of the National Institute of Information and Communications Technology, Japan. Subjects learned conditioned associations between different visual cues (abstract colored images presented on a computer screen) and brief painful heat stimuli delivered either to the left forearm, the right forearm, or not at all. Ultra-brief painful heat stimuli at 55°C were delivered through two contact heat-evoked potential stimulators (CHEPS; Medoc Pathway) to the subject’s left or right inner forearm.

### Physiological Measurement and Analysis

Physiological signals were continuously recorded using MRI-compatible BrainAmp ExG MR System with specialized electrodes and sensors (Brain Products; see Figure S1). Off-line processing and analysis were implemented in MATLAB7 (The MathWorks).

SCRs were assessed as the peak-to-peak amplitude difference in a time window of 0.5–4.5 s after cue onset (pain-omitted trials) and 0.5–5.5 s (pain trials). Raw SCR magnitudes were square root transformed for normalization and scaled to individual subject’s mean-square-root-transformed US response [7, 33]. Upper-limb EMG recordings were taken from the brachioradialis and biceps-brachii muscles on both arms. MRI artifacts were removed by using a custom-made filtering program [34]. The resultant EMG signals were band-pass filtered at 10–150 Hz, full wave rectified, and baseline adjusted. The signals from 1-s CS-US interval were sectioned out and sorted according to trial types for further analysis. Moreover, conditioned EMG response (CR) was defined as where ISI EMG activity reached 30% of the EMG maximum of that trial, staying above that with a minimum duration of 200 ms and a minimum integral of 1 mV/ms [29]. The percentage of EMG CR incidence was averaged across left and right. Facial EMG (corrugator muscle) and heart rate were collected in behavioral study only (see Supplemental Experimental Procedures). Due to hardware constraint, SCRs were recorded on left side only, as there is no definitive evidence of laterality difference between electrodermal activity recorded on left or right hand [35].

### Computational Model Analysis

We constructed reinforcement learning models, fitted trial-by-trial model value/associability to SCR data for parameter estimation and model comparison, and then used obtained learning signals to probe brain activity [7, 8, 33]. In this way, the brain responses are specifically related to the behaviorally fitted learning model. These models can be used to test competing hypotheses about the neural representation of preparatory (i.e., laterality non-specific) and consummatory (i.e., laterality specific) learning processes.

#### Standard Temporal Difference Model

This model is the simple “real-time” instantiation of the Rescorla-Wagner (RW) model [36]. The value *V* of trial *n* + 1 for a given cue *j* is updated based on the value of current trial *n* and the prediction error, difference between current value *V*_*j*_, and outcome stimulus value *R* at trial *n*, weighted by a constant learning rate α:

$$V_{j}\left(n+1\right)=V_{j}\left(n\right)+\alpha \cdot \left(R\left(n\right)-V_{j}\left(n\right)\right),$$

where the learning rate α (0 ≤ α ≤ 1) is a free parameter.

#### Hybrid Temporal Difference Model

The hybrid model combines both RW and Pearce-Hall (PH) models, where the RW rule is used for error-driven value update and PH associability is used as a dynamic learning rate for RW to modulate predictive learning [7]. The value of associability decreases if the conditioned stimuli become correctly predictive of the stimuli outcome [37]. The values of hybrid model were updated as follows:

$$V_{j}\left(n+1\right)=V_{j}\left(n\right)+\kappa \cdot \alpha_{j}\left(n\right)\cdot \left(R\left(n\right)-V_{j}\left(n\right)\right)$$

$$\alpha_{j}\left(n+1\right)=\eta \cdot \left|R\left(n\right)-V_{j}\left(n\right)\right|+\left(1-\eta \right)\cdot \alpha_{j}\left(n\right),$$

where free parameters α_0_ (initial associability; 0 ≤ α_0_ ≤ 1), κ(0 ≤ κ ≤ 1), and η(0 ≤ η ≤ 1) are determined by fitting to behavioral data.

Assuming the preparatory learning system cannot distinguish lateralized outcomes, then *R*(*n*) = 1 for all pain trials regardless of laterality. Whereas the consummatory learning system tracked outcomes ipsilateral to its side only, ignoring the opposite side, then for the left system, *R*(*n*) = 1 for left pain or *R*(*n*) = 0 for both right pain and no pain and vice versa for the right system.

For individual session, the free parameters were optimized by maximizing likelihood for individual subject’s sequence of SCRs, modeled as the normal distribution around a mean determined by the scaled predicted value (or associability or the sum of both), computed by the model on that trial, plus a constant error term with a distribution variance [7]. To avoid contamination by pain over CS-predictive responses, only SCRs of no pain (i.e., unreinforced) trials were fitted, but all trials were used in the computation of value and associability. We obtained population free parameters using a hierarchical-model-fitting approach for subsequent imaging analysis [38]. Bayesian information criterion (BIC) value was calculated for each model with optimal individual parameters to quantitatively compare goodness of fit (Table S2).

### fMRI Data Analysis

fMRI imaging data were acquired on a 3T Siemens Magnetom Trio scanner with Siemens standard 12-channel phased array head coil. Functional images were collected using a single-shot gradient echo EPI sequence (repetition time [TR] = 2,500 ms; echo time [TE] = 30 ms; field of view = 240 mm; flip angle = 80°). Thirty-seven contiguous oblique-axial slices (3.75-mm voxels) parallel to the AC-PC line were acquired. Whole-brain high-resolution T1-weighted structural images were obtained. Preprocessing of imaging data were conducted using SPM8 following standard procedures (Wellcome Trust Center for Neuroimaging; http://www.fil.ion.ucl.ac.uk/spm/).

We conducted a parametric analysis, in which the computational model generated learning signal regressors parametrically modulated stick functions at the time of CS (visual cue) and US (pain outcome) presentation for each trial [39]. The best-fitting hybrid model from the SCR-based analysis was used to generate the following regressors with population free parameters: *at outcome time*: (1) preparatory associability α_*general*_; (2) left-sided consummatory associability α_*left*_; and (3) right-sided associability α_*right*_; *at cue and outcome time* (i.e., “full” prediction error as a biphasic response): (4) preparatory prediction error *VD*_*general*_; (5) left-sided predicted error series *VD*_*left*_; and (6) right-sided prediction error series *VD*_*right*_; *regressors of no interest*: (7) and (8) left/right pain delivery and (9) motion parameters (×6) from affine realignment in preprocessing.

All these regressors were compiled into one single GLM for first-level analysis for individual subject in SPM8. Resulting contrasts were used in second-level one-sample t tests to make population inference (Figure 3). Small volume correction (SVC) for multiple comparison was conducted within anatomically defined 8-mm-diameter spherical masks built around hypothesized structure coordinates of the amygdala, ventral putamen, and cerebellum (Table S1).

Functional ROI analysis of the cerebellum was conducted using SUIT atlas [40]. Masks of the cerebellum were created using T1-weighted structural scans for each subject, spatially normalized to the SUIT template. Resultant contrasts from first-level analyses were then resliced into SUIT atlas space using previously generated SUIT normalization parameters. Spatial smoothing of the functional data was omitted in order to avoid contaminating activation from the visual cortex. The SUIT probabilistic MRI atlas of human cerebellum was used to locate cerebellar lobules [41]. In addition, post hoc analyses of all ROIs were conducted by extracting beta estimates for each subject from the functional clusters of interest as they appear in given contrasts using MarsBaR toolbox (http://marsbar.sourceforge.net/). They were then averaged across subjects according to model or trial types without parametric modulation (Figures S3G and S3H).

## Author Contributions

S.Z., G.G., T.R., and B.S. designed the experiment. S.Z. and H.M. performed the experiment. S.Z., H.M., G.G., and B.S. analyzed the data. All authors contributed to the writing of the manuscript.

## Supplemental Information

Document S1. Figures S1–S3, Tables S1 and S2, and Supplemental Experimental Procedures

Document S2. Article plus Supplemental Information

## References

[1] LeDoux J.E. (2014). Coming to terms with fear. Proc. Natl. Acad. Sci. USA.

[2] Gerber B., Yarali A., Diegelmann S., Wotjak C.T., Pauli P., Fendt M. (2014). Pain-relief learning in flies, rats, and man: basic research and applied perspectives. Learn. Mem..

[3] Wager T.D., Atlas L.Y. (2015). The neuroscience of placebo effects: connecting context, learning and health. Nat. Rev. Neurosci..

[4] Phelps E.A., LeDoux J.E. (2005). Contributions of the amygdala to emotion processing: from animal models to human behavior. Neuron.

[5] Seymour B., O’Doherty J.P., Dayan P., Koltzenburg M., Jones A.K., Dolan R.J., Friston K.J., Frackowiak R.S. (2004). Temporal difference models describe higher-order learning in humans. Nature.

[6] Daum I., Schugens M.M., Ackermann H., Lutzenberger W., Dichgans J., Birbaumer N. (1993). Classical conditioning after cerebellar lesions in humans. Behav. Neurosci..

[7] Li J., Schiller D., Schoenbaum G., Phelps E.A., Daw N.D. (2011). Differential roles of human striatum and amygdala in associative learning. Nat. Neurosci..

[8] Boll S., Gamer M., Gluth S., Finsterbusch J., Büchel C. (2013). Separate amygdala subregions signal surprise and predictiveness during associative fear learning in humans. Eur. J. Neurosci..

[9] Holland P.C., Gallagher M. (2006). Different roles for amygdala central nucleus and substantia innominata in the surprise-induced enhancement of learning. J. Neurosci..

[10] Holland P.C., Gallagher M. (1993). Amygdala central nucleus lesions disrupt increments, but not decrements, in conditioned stimulus processing. Behav. Neurosci..

[11] Johansen J.P., Tarpley J.W., LeDoux J.E., Blair H.T. (2010). Neural substrates for expectation-modulated fear learning in the amygdala and periaqueductal gray. Nat. Neurosci..

[12] McHugh S.B., Barkus C., Huber A., Capitão L., Lima J., Lowry J.P., Bannerman D.M. (2014). Aversive prediction error signals in the amygdala. J. Neurosci..

[13] Blair H.T., Huynh V.K., Vaz V.T., Van J., Patel R.R., Hiteshi A.K., Lee J.E., Tarpley J.W. (2005). Unilateral storage of fear memories by the amygdala. J. Neurosci..

[14] Apergis-Schoute A.M., Schiller D., LeDoux J.E., Phelps E.A. (2014). Extinction resistant changes in the human auditory association cortex following threat learning. Neurobiol. Learn. Mem..

[15] Balleine B.W., Killcross S. (2006). Parallel incentive processing: an integrated view of amygdala function. Trends Neurosci..

[16] Prévost C., McCabe J.A., Jessup R.K., Bossaerts P., O’Doherty J.P. (2011). Differentiable contributions of human amygdalar subregions in the computations underlying reward and avoidance learning. Eur. J. Neurosci..

[17] Bingel U., Gläscher J., Weiller C., Büchel C. (2004). Somatotopic representation of nociceptive information in the putamen: an event-related fMRI study. Cereb. Cortex.

[18] Torrecillos F., Albouy P., Brochier T., Malfait N. (2014). Does the processing of sensory and reward-prediction errors involve common neural resources? Evidence from a frontocentral negative potential modulated by movement execution errors. J. Neurosci..

[19] Roy M., Shohamy D., Daw N., Jepma M., Wimmer G.E., Wager T.D. (2014). Representation of aversive prediction errors in the human periaqueductal gray. Nat. Neurosci..

[20] Moulton E.A., Schmahmann J.D., Becerra L., Borsook D. (2010). The cerebellum and pain: passive integrator or active participator?. Brain Res. Brain Res. Rev..

[21] Saab C.Y., Willis W.D. (2003). The cerebellum: organization, functions and its role in nociception. Brain Res. Brain Res. Rev..

[22] Kelly R.M., Strick P.L. (2003). Cerebellar loops with motor cortex and prefrontal cortex of a nonhuman primate. J. Neurosci..

[23] Dimitrova A., Kolb F.P., Elles H.-G., Maschke M., Forsting M., Diener H.C., Timmann D. (2003). Cerebellar responses evoked by nociceptive leg withdrawal reflex as revealed by event-related FMRI. J. Neurophysiol..

[24] Ploghaus A., Tracey I., Gati J.S., Clare S., Menon R.S., Matthews P.M., Rawlins J.N.P. (1999). Dissociating pain from its anticipation in the human brain. Science.

[25] Singer T., Seymour B., O’Doherty J., Kaube H., Dolan R.J., Frith C.D. (2004). Empathy for pain involves the affective but not sensory components of pain. Science.

[26] Stoodley C.J., Schmahmann J.D. (2009). Functional topography in the human cerebellum: a meta-analysis of neuroimaging studies. Neuroimage.

[27] Timmann D., Baier P.C., Diener H.C., Kolb F.P. (2000). Classically conditioned withdrawal reflex in cerebellar patients. 1. Impaired conditioned responses. Exp. Brain Res..

[28] Lavond D.G., Steinmetz J.E. (1989). Acquisition of classical conditioning without cerebellar cortex. Behav. Brain Res..

[29] Thieme A., Thürling M., Galuba J., Burciu R.G., Göricke S., Beck A., Aurich V., Wondzinski E., Siebler M., Gerwig M. (2013). Storage of a naturally acquired conditioned response is impaired in patients with cerebellar degeneration. Brain.

[30] Schultz W., Dickinson A. (2000). Neuronal coding of prediction errors. Annu. Rev. Neurosci..

[31] Pearce J.M., Montgomery A., Dickinson A. (1981). Contralateral transfer of inhibitory and excitatory eyelid conditioning in the rabbit. Q. J. Exp. Psychol. Sect. B.

[32] Betts S.L., Brandon S.E., Wagner A.R. (1996). Dissociation of the blocking of conditioned eyeblink and conditioned fear following a shift in US locus. Anim. Learn. Behav..

[33] Schiller D., Levy I., Niv Y., LeDoux J.E., Phelps E.A. (2008). From fear to safety and back: reversal of fear in the human brain. J. Neurosci..

[34] Ganesh G., Franklin D.W., Gassert R., Imamizu H., Kawato M. (2007). Accurate real-time feedback of surface EMG during fMRI. J. Neurophysiol..

[35] Dawson M.E., Schell A.M., Filion D.L., Cacioppo J.T., Tassinary L.G., Berntson G. (2007). The electrodermal system. Handbook of Psychophysiology.

[36] Rescorla R.A., Wagner A.R., Black A.H., Prokasy W.F. (1972). A theory of Pavlovian conditioning: variations in the effectiveness of reinforcement and nonreinforcement. Classical Conditioning II: Current Research Theory.

[37] Pearce J.M., Hall G. (1980). A model for Pavlovian learning: variations in the effectiveness of conditioned but not of unconditioned stimuli. Psychol. Rev..

[38] Daw N.D., Delgado M.R., Phelps E.A., Robbins T.W. (2011). Trial-by-trial data analysis using computational models. Decision Making, Affect, and Learning: Attention and Performance XXIII.

[39] O’Doherty J.P., Hampton A., Kim H. (2007). Model-based fMRI and its application to reward learning and decision making. Ann. N Y Acad. Sci..

[40] Diedrichsen J. (2006). A spatially unbiased atlas template of the human cerebellum. Neuroimage.

[41] Diedrichsen J., Balsters J.H., Flavell J., Cussans E., Ramnani N. (2009). A probabilistic MR atlas of the human cerebellum. Neuroimage.

