# Supplementary material for: Dissociable Learning Processes Underlie Human Pain Conditioning
Source: Curr Biol. 2016 Jan 11;26(1):52–8. doi: 10.1016/j.cub.2015.10.066 (PMC4712170; doi:10.1016/j.cub.2015.10.066)
Supplement: Document S1. Figures S1–S3, Tables S1 and S2, and Supplemental Experimental Procedures [file mmc1.pdf]

Current Biology

Supplemental Information

Dissociable Learning Processes Underlie Human Pain Conditioning

Suyi Zhang, Hiroaki Mano, Gowrishankar Ganesh, Trevor Robbins, and Ben Seymour

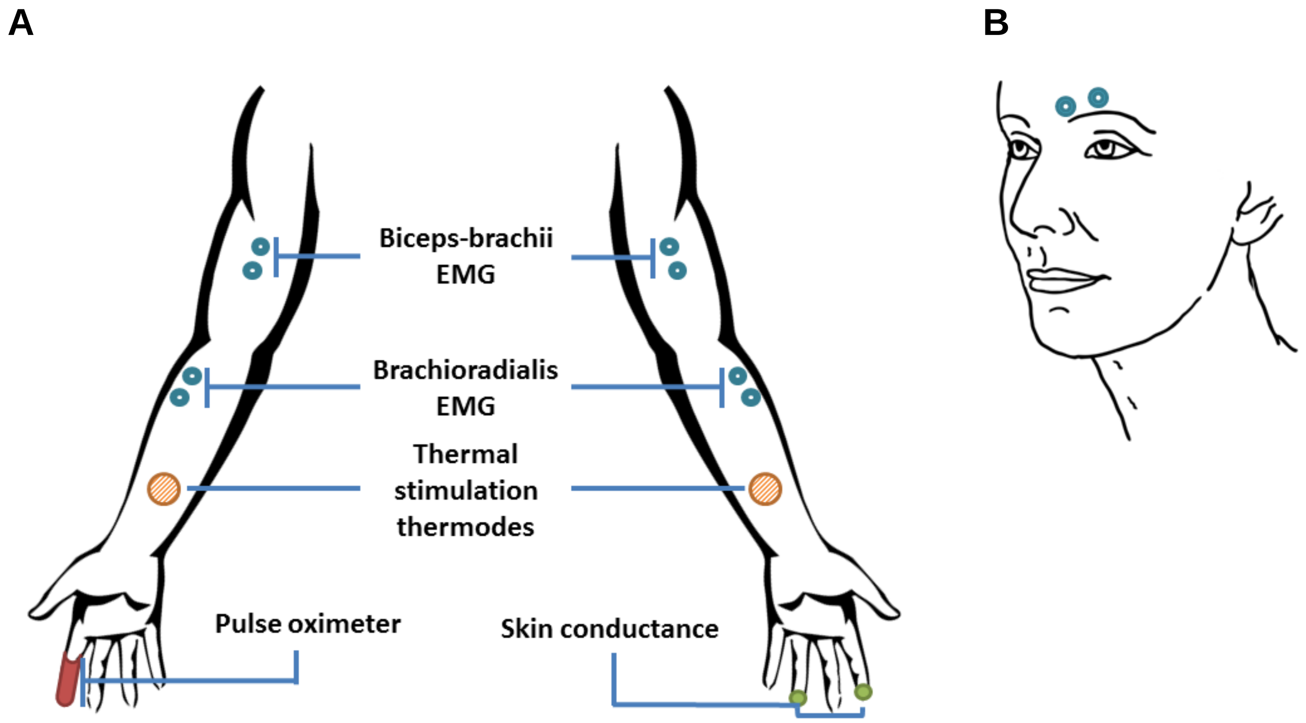

Figure S1. Recording and stimulating apparatus placement. Related to Experimental Procedures.

(A) Diagram showing placement of bilateral upper-limb EMG electrodes (brachioradialis and biceps-brachii), pulse oximeter (heart rate), skin conductance electrodes, and stimulation thermodes.

(B) Placement of facial EMG (corrugator) electrodes.

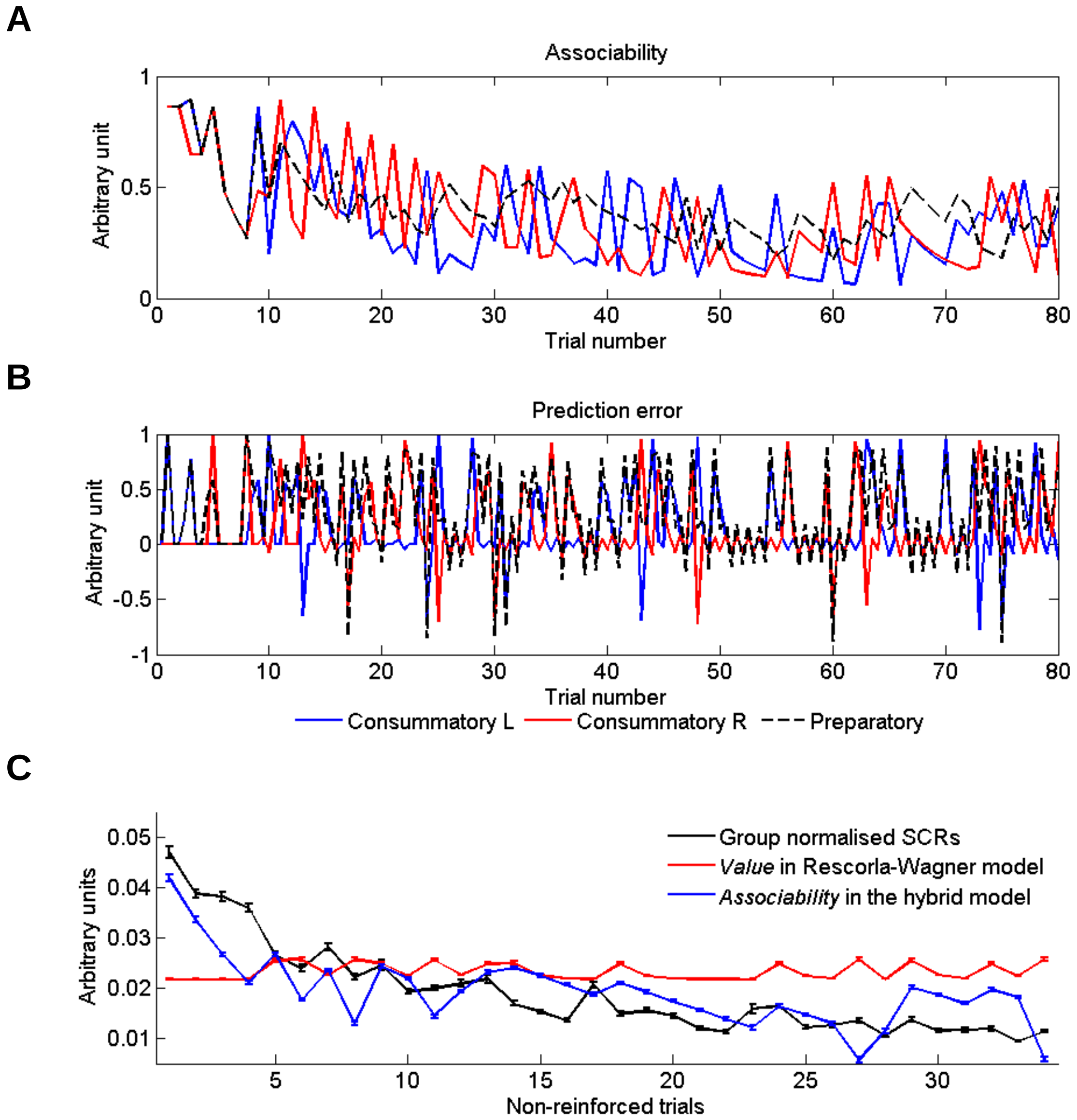

Figure S2. Exemplary Associability and Prediction error plot (in 1 session). Related to Figure 2.

(A) Associability traces.

(B) Prediction error traces, for consummatory left and right, and preparatory responses, estimated by hybrid model. Population free parameters from SCR fitting were used to determine model output.

(C) Trial-by-trial fitting to group normalized SCRs of non-reinforced trials.

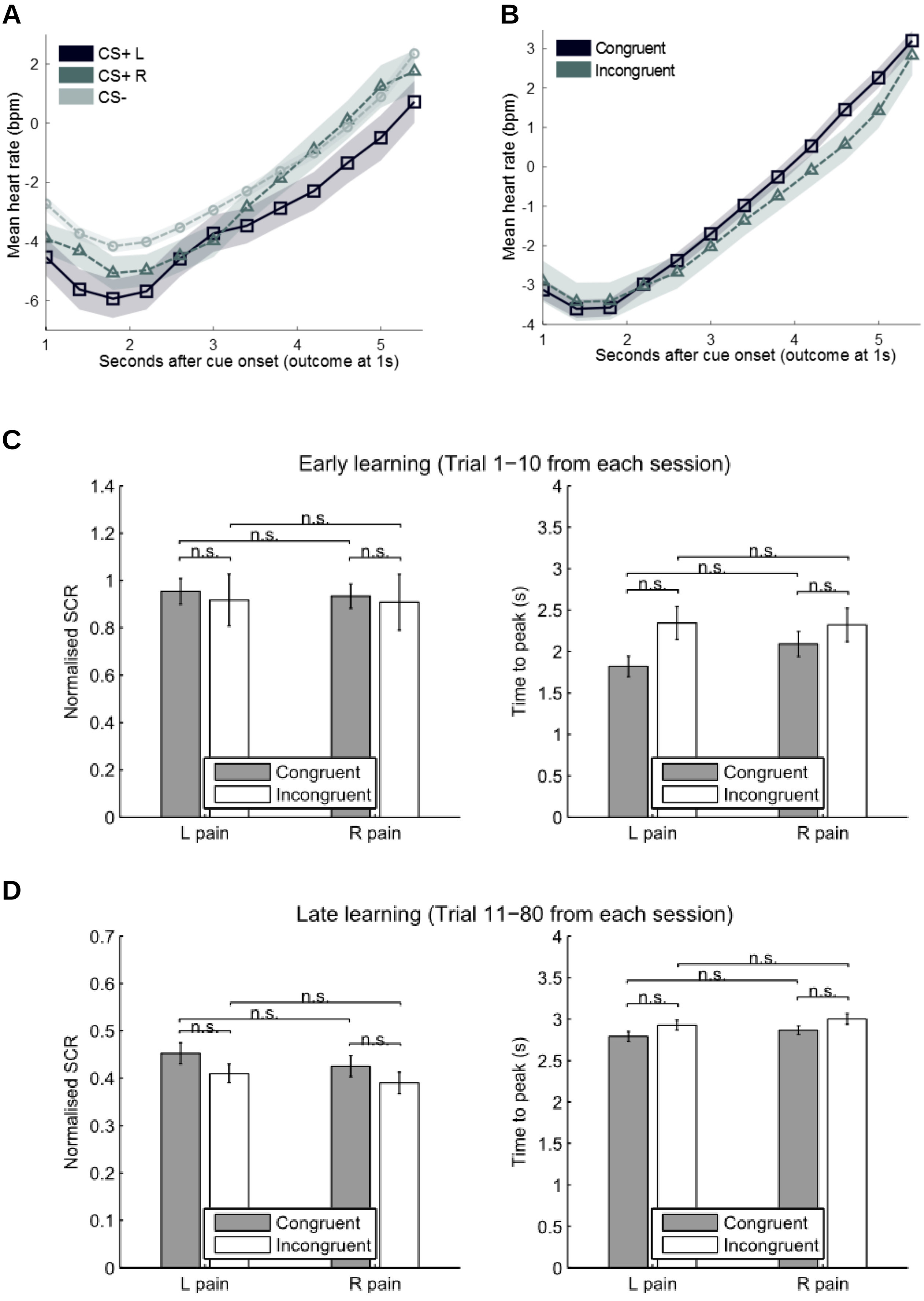
A
CS+ L
CS+ R
CS-
Mean heart rate (bpm)
Seconds after cue onset (outcome at 1s)
B
Congruent
Incongruent
Mean heart rate (bpm)
Seconds after cue onset (outcome at 1s)
C
Early learning (Trial 1−10 from each session)
Normalised SCR
Time to peak (s)
n.s.
Congruent
Incongruent
L pain
R pain
D
Late learning (Trial 11−80 from each session)
Normalised SCR
Time to peak (s)
n.s.
Congruent
Incongruent
L pain
R pain

E

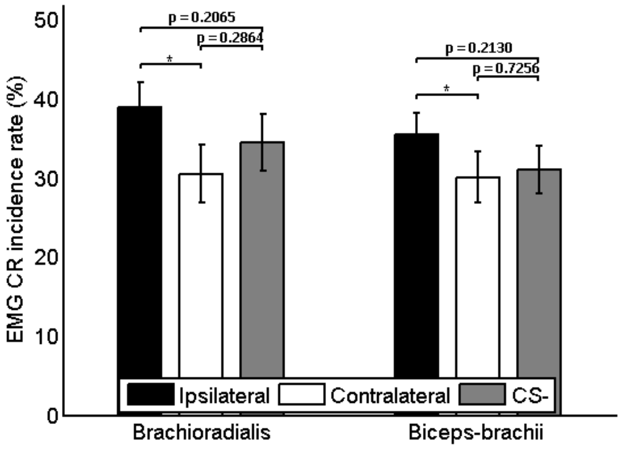

F

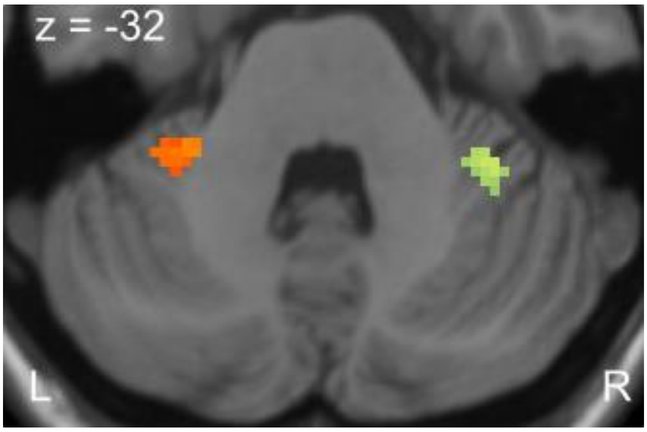

G

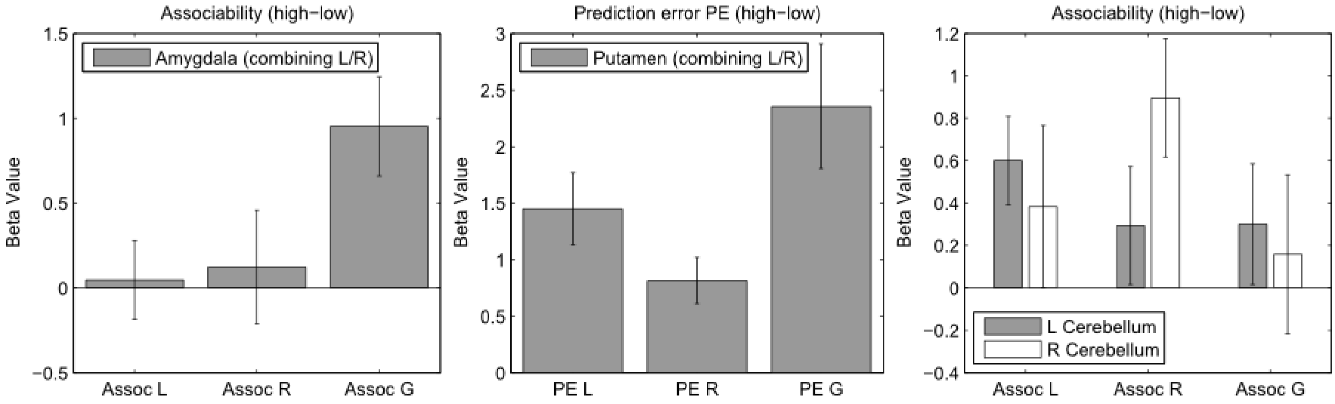

H

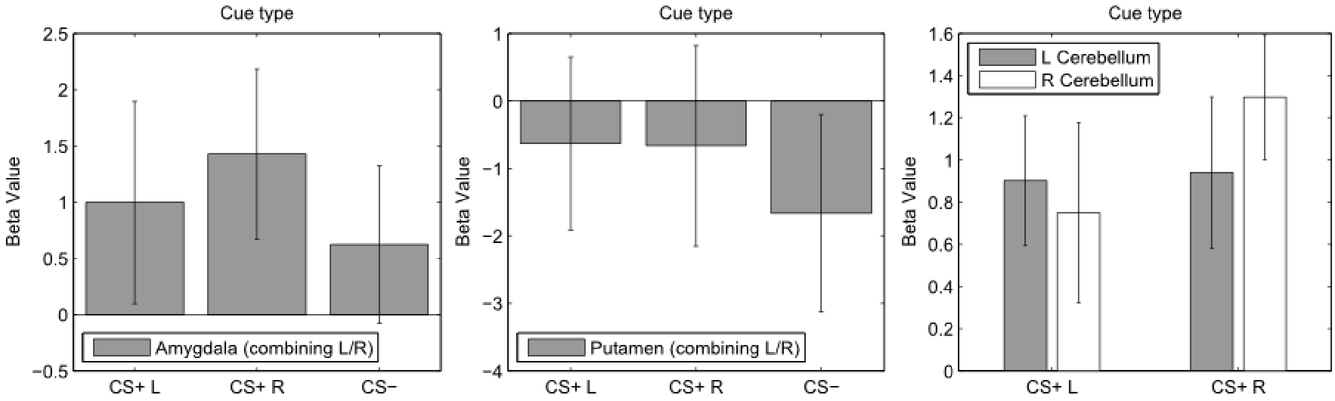

Figure S3. Additional results (heart rate, skin conductance responses, upper-arm EMG, post-hoc analysis for fMRI regions of interest). Related to Figure 2 and 3.

(A) Heart rate changes of ‘unreinforced’ trials in time window 1-5.5s after cue onset (behavioral, 2 sessions). CS+ L/R show no significant difference ($p>0.4$ for all time points), but both significantly different with CS- ($p<0.05$: vs. CS+ L all time points, vs. CS+ R 1-3s), with more pronounced deceleration (1-3s) and acceleration (4-5.5s);

(B) Heart rate changes in pain trials (behavioral, 2 sessions). Incongruent prediction trials (gray) showed less pronounced acceleration over congruent trials (black), but both followed a similar pattern (all sample points not significant, $p>0.1$); This is consistent with previous results when subjects were given novel (surprising) aversive stimuli [S1], and raises the possibility that some autonomic responses may not entirely blind to the laterality of pain, and hence may not be purely a preparatory response. This possibility needs further investigation.

(C) Additional analysis on SCR were conducted by separating trials into early (trials 1-10) and late (trials 11-80) learning periods in each session (scanning, 3 sessions). SCRs were pooled according to congruent and incongruent predictions, separately for L and R pain trials. Early learning trials showed no significant laterality differences in either SCR magnitude (measured as peak-to-peak magnitude at time window 0.5-5.5s after CS onset), or the time taken to rise to peak SCR (measured as the time difference from minimum to maximum SCR magnitude).

(D) Late learning trials also showed no significant laterality differences in either SCR magnitude or rise time to peak.

(E) Mean upper-arm EMG conditioned response (CR) incidence (scanning, 3 sessions). CR incidence was significantly higher for ipsilateral CS trials, while the level is comparable for the contralateral CS and CS- groups. The relatively high CR incidence of contralateral CS and CS- trials could be a result of EMG activity associated with general preparatory withdrawal (e.g. leaning backwards), which could be induced by the probabilistic paradigm.

(F) Post-hoc analysis of cerebellum ROIs. Trials were split into high and low categories, for associabilities calculated by L/R consummatory systems respectively. Clusters shown were activations from associability category high minus low, with no parametric modulation: Assoc L (orange, peak coordinates [-28 -36 -32], T=3.38), Assoc R (green, peak coordinates [30 -36 -28] T=3.97), both visualised at $p<0.005$;

(G) Beta values extracted from ROIs identified (bilateral amygdala, putamen, and cerebellum) from trial category high minus low (Associability or prediction errors, with no parametric modulation), for both lateralised consummatory systems (Assoc/PE L/R) and general preparatory system (Assoc/PE G). Amygdala and putamen ROIs showed higher values for general system outputs (G), while cerebellum ROIs showed differential high value for ipsilateral system outputs (L/R).

(H) Beta values extracted from ROIs identified and pooled according to CS types at US onset time, with no parametric modulation. Amygdala and putamen ROIs showed similarly high values for both CS+ L/R, but lower value for CS-. R cerebellum ROI showed higher response for ipsilateral CS+, however, L cerebellum failed to show significant difference. It is important to note that this categorical trial-based analysis doesn't capture the dynamics of learning inherent in the parametrically modulated and behavioural fit learning model, this being the reason for the relatively broad error bars.

Data are represented as mean ± SEM. *$p < 0.05$; n.s., not significant. Assoc: associability.

Supplemental Tables

Table S1. Small volume correction (SVC) for multiple comparison within anatomically defined 8mm spherical masks of hypothesized structure coordinates (We did not find evidence of consummatory prediction errors on either side – see text). Related to Figure 3.					
	peak p (FWE-corr)	cluster size	T statistics	Z statistics	x, y, z {mm}
**Associability (preparatory)**					
Amygdala (L)	0.089	13	2.96	2.57	-28 -6 -18
	0.112		2.78	2.44	-32 -4 -16
Amygdala (R)	0.038	36	3.58	2.97	30 -4 -18
**Prediction error (preparatory)**					
Ventral putamen (L)	0.002	43	5.77	4.06	-32 2 2
	0.003	43	5.67	4.02	-24 -4 -6
Ventral putamen (R)	0.003	91	5.50	3.95	26 -4 -6
	0.014		4.49	3.48	34 -4 -2
**Associability (consummatory left)**					
Cerebellum (L)	0.017	15	4.19	3.32	-28 -40 -30
**Associability (consummatory right)**					
Cerebellum (R)	0.011	7	4.55	3.51	32 -38 -30

Table S2. Goodness of fit to SCRs for individual models (scanning, 3 sessions 15 subjects, 34 non-reinforced trials per session per subject. V: model values, α: associabilities). Related to Figure 2.

**Model**	**Mean BIC**	**No. of free parameters**
RW (V)	-193.34	4
Hybrid (V)	-186.69	6
Hybrid (α)	-198.73	6
Hybrid (V + α)	-197.36	7

Supplemental Experimental Procedures

Subjects

Fifteen healthy subjects (1 female) participated in the imaging study, and twelve subjects (3 females) in an initial behavioral study. Both behavioral and imaging studies had the same experimental design, but differed in the physiological data that was measured. All subjects were right-handed, had normal or corrected to normal vision, and were free of pain or pain medication. All subjects gave informed consent prior to participation, and the study was approved by the Ethics and Safety committee of the National Institute of Information and Communications Technology, Japan.

Experimental design

Subjects participated in a Pavlovian first-order delay conditioning experiment in which they learned conditioned associations between different visual cues (abstract colored images presented on a computer screen) and brief painful heat stimuli delivered either to the left forearm, the right forearm, or not at all (see 'stimulation delivery and apparatus' for stimuli details). There were three sessions in the experiment, each consisting of 80 trials lasting approximately 12 minutes.

On each trial, a visual cue (the conditioned stimulus, CS) was displayed in the middle of a neutral grey background on a computer screen for 1 second. The disappearance of the cue after 1s was immediately followed by delivery of a lateralized (i.e. either left or right) thermal pain stimulus (the unconditioned stimuli, US), or no stimulus at all. The next trial was triggered to start after a 7-9s (mean=8s) inter-trial interval (Figure 1B). There were three types of cue in total, denoted as cue CS+ left, CS+ right, and CS- (Figure 1A). All three cue-outcome contingencies were probabilistic. Throughout the experiment, CS+ L predicted the subsequent delivery of left sided pain with 70% probability, right sided pain 15%, or no pain with 15% probability. CS+ R had a similar pattern to CS+ L, except the laterality was reversed (i.e. 70% of the time with a right pain etc.). The CS- cue was followed by no pain 70% of the time, with left and right pain probabilities at 15% each. In the total of 80 trials per session, half were CS+ trials (20 CS+ L and 20 CS+ R), and the other half were CS- trials. Trials were presented in a pseudo-random order.

An important feature of the paradigm design is the use of a relatively short CS-US (inter-stimulus) interval (ISI). In many human Pavlovian conditioning experiments, this is typically set at about 3-4s [S2–5], as opposed to 1s in our design. However, very short ISIs allow well timed predictions of pain to be made, which allows much easier detection of time-locked conditioned electromyography (EMG) responses. This is more similar to eye-blink conditioning studies (e.g., in rabbits), where the optimal interval is typically 400-1000ms [S6]. Such rapid intervals should therefore be optimal for detecting 'reflexive' limb activity. Although it is possible that conscious deliberative movements could be part of an explicit CS-US contingent response (which is better thought of as reflecting 'contingency awareness' than true conditioning), sub-second time-locked responses within a 1s CS-US interval is firmly in the domain of Pavlovian responding: indeed Kaulich and colleagues have elegantly shown the progressive acquisition of EMG responses at similar latencies [S7].

The only other difference between the initial behavioral study and the imaging study was the former had two instead of three sessions. There were no changes in terms of paradigm, but different physiological data were recorded in each (see below). There were no specific task instructions for subjects, except requiring them to pay attention to the screen.

We briefly note here that in fMRI-based conditioning experiments, short ISIs can be a problem when discriminating predictive signals (e.g. conditioned value or temporal prediction error) from outcome signals, because of the slow blood oxygenation level dependent (BOLD) response. However, this is not an issue here, because our hypothesis is based on discriminating laterality, and not testing *de novo* predictions about the precise nature of the signal in, for example, the striatum, which has been well characterized in recent years [S8]. We discuss the nature of computational models below, but note that identification of 'associability' signals is not problematic in short-ISI designs, since this signal reflects an uncertainty signal that pervades the whole of the CS-US trial.

Stimulation delivery and apparatus

We used thermal pain as the unconditioned stimulus. Importantly, this selectively activates thermally sensitive a-delta and c-fiber afferents, and so can be regarded as nociceptive specific. This is important, as we did not want to activate other sensory fibers, especially proprioceptive afferents, which play a role in non-pain sensorimotor feedback learning, known to be dependent on the cerebellum [S9].

Painful thermal stimuli were delivered through two contact heat-evoked potential stimulators (CHEPS, Medoc Pathway, Israel). The CHEPS thermode is capable of delivering ultra-brief heat stimuli at maximum 55° C with a rising rate of 70° C/s, followed by rapid cooling at 40° C/s back to baseline temperature at 32° C. The thermodes were attached to the subject's skin surface above the wrists on the inner forearms bilaterally using Velcro straps. The maximum temperature

deliverable at 55° C was selected for all subjects after confirming it was within their tolerance and reached the intensity level of 'painful'.

To minimize noise artifact, each of the two CHEPS stimulators was passed through the custom MEDOC filter built into the aluminum filter panel on the wall between the control room and scanning room. In the scanning room, this attached to each fMRI compatible thermode which ran along the scanning room floor to the subject.

Behavioral and psychophysical measurement

Psychophysical signals were continuously measured and recorded for the duration of experiment using MRI compatible BrainAmp ExG MR System (Brain Products, Munich, Germany, Figure S1). All signals were collected at sampling rate 5000Hz. Off-line processing and analysis were implemented in MATLAB7 (The MathWorks Inc., Natick, MA, USA). The subjects in the behavioral study were in a sitting position with their forearms naturally resting on a table in front, while those in the imaging study lay supine with both arms positioned on either side in the scanner.

In the initial behavioral study, we report data below from heart rate and facial EMG, which was not recorded in the imaging experiment. We initially attempted to collect skin conductance responses (SCR) data but this was abandoned because of a technical failure. The main purpose of the behavioral experiment was to optimize different limb EMG montages for the imaging study (hence this data is not reportable, as it involves a range of exploratory montages). In the imaging experiment we used the optimized limb EMG, and SCR, as our main behavioral outcome measures.

Skin conductance responses (SCR, imaging study only)

There are two ways to infer conditioning from skin conductance responses (SCRs). First, it is possible to look at the anticipatory SCR following the conditioning cue (CS), which is typically done during the trials in which pain is omitted, because of the slow response time of the SCR (time-to-peak is usually several seconds). This is sometimes referred to as an indirect measure of conditioning [S10]. Alternatively, one can look at the SCR to the pain itself, to see how it is modified by the whether or not it was predicted - a so-called direct measure of conditioning. Both measures are utilized here. SCRs were measured with BrainAmp Ag/AgCl sintered MR electrodes, filled with skin conductance electrode paste, and attached to the distal phalanges of the second and fourth fingers on subject's left hand. SCR amplitude is a well-studied behavioral index of threat / fear in aversive conditioning studies [S5, 11].

The level of SCR was assessed for pain-omitted trials by calculating the peak-to-peak amplitude difference in a time window of 0.5-4.5s after cue onset. For pain trials, the time window was prolonged to 0.5-5.5s (because the pain occurs 1s after the cue). Raw SCR magnitudes were square root transformed for normalization, and then scaled to individual subject's mean square root transformed US response [S11]. Paired t-tests were used for statistical testing.

In the computational model based analysis, we used the Bayesian information criterion (BIC) to compare goodness of fit of both Rescorla-Wagner and hybrid models to behavioral SCR sequences (Table S2). The associability time series α from hybrid model fit the SCRs better than other models, giving the lowest BIC value, despite having been penalized for having a larger number of free parameters than RW model. This can be easily visualized by inspecting the comparison plots in Figure 2G and Figure S2C. The results were consistent with the findings of Li et al. [S5], suggesting fitting SCR data for determining free parameters in the hybrid model was valid.

Facial EMG (behavioral study only)

Surface EMG recordings were taken from the corrugator muscles on the left side with electrodes attached to skin over the eyebrow, lateral to the midline at a slightly oblique angle (Figure S1B). The corrugator is responsible for furrowing of the brow, and has been used for assessing nociception during anesthesia and in emotional conditioning experiments [S12]. The reflex-like conditioned response of spontaneous facial expressions are characterized as symmetrical and synchronized, hence unilateral EMG measurement of corrugator was considered sufficient [S13]. EMG traces of trials were visually inspected and suspected eye-blink contaminated trials were excluded from analysis. For details of off-line processing see 'Upper-limb EMG'.

Heart rate (behavioral study only)

Heart rate was estimated using recordings from a pulse oximeter (BrainAmp, Brain Products, Munich, Germany) placed on the forefinger of the subject's right hand. Due to hardware constraints this recording was only collected in the behavioral study. The baseline heart rate was estimated by averaging a 15s recording after the experiment concluded. Subjects' heart rate was estimated from the continuous recordings for the duration of the experiment. Cardiac deceleration is thought to be an index of perceptual processing of sensory information through parasympathetic activity, and unpleasant stimuli are associated with more pronounced deceleration [S1]. Cardiac acceleration has been interpreted as evidence of mobilization for avoidance, and is related to muscle preparation [S14].

Upper-limb EMG (imaging study only)

Upper-limb EMG recordings were taken from the brachioradialis and biceps-brachii muscles on both arms (Figure S1A). Pairs of MR-compatible EMG electrodes (Brain Products, Munich, Germany) were placed on the muscle belly parallel to the muscle fibers, 2cm apart. Ground electrodes were placed on the lateral epicondyle of the elbow bilaterally. Brachioradialis was located by palpating the muscle mass distal to the elbow while resisting elbow flexion in thumb up position, and biceps-brachii was located by palpating the muscle mass dorsal to the elbow during isometric forearm flexion. The overlaying skin was cleaned with alcohol wipes.

Raw EMG signals were processed offline using MATLAB. MRI artefacts were removed by using a custom-made filtering program [S15]. The resultant EMG signals were band-pass filtered between 10-150Hz and full wave rectified. The signals from 1s CS-US interval were sectioned out for all trials, and sorted according to ipsilateral (CS+ cue predictive of the side to receive pain) and contralateral (CS+ cue predictive of the opposite side) trials for further analysis. For plotting and statistical comparison the signal traces were down-sampled to 100Hz.

To assess the overall difference in EMG traces during the 1s CS-US interval for ipsilateral and contralateral trials, baseline EMG was first subtracted from individual muscle for all trials, then the change in EMG trace from ipsilateral brachioradialis and biceps-brachii were added for amplification. Resultant EMG traces were averaged across subjects according to trial types. Paired t-tests were used for trace comparison in specific time points.

To assess conditioned responses trial by trial, mean conditioned response (CR) incidence of processed upper-arm EMG during the 1s CS-US interval was calculated using methods previously employed in identification of conditioned eye-blink EMG responses [S16]. Conditioned EMG response was defined as where ISI EMG activity reached 30% of the EMG maximum of that trial, staying above that with a minimum duration of 200ms, and a minimum integral of 1mV/ms. The percentage of EMG CR incidence were calculated for ipsilateral / contralateral trials respectively, and were averaged across left and right. Wilcoxon rank sum test was used for statistical tests. The null hypothesis rejection level for all statistical tests was at $p<0.05$.

Given the limited degree of freedom in a sitting (behavioral study) and supine position (imaging study), we expected the specific conditioned responses to lead to arm flexion. We therefore chose to monitor brachioradialis and biceps-brachii because they are relatively large flexor muscles of the upper limb that are easy to locate and usually offer good signal to noise ratio in the EMG measurements. Furthermore, biceps-brachii being a bi-articulate muscle, the activation of these two muscles covers a range of upper-limb motions including forearm flexion, supination, shoulder flexion and elbow flexion [S12]. There are previous examples of the study of conditioned withdrawal responses using surface EMG from the lower limbs [S7, 17], but we are not aware of prior examples in upper limbs.

Because of the close physical proximity of the EMG electrodes and the thermal stimulator thermode on the forearm, EMG signals become contaminated by electrical noise after stimulation, and therefore it was not possible to reliably interpret direct EMG responses following pain stimulation. However, the anticipatory EMG provided clear and sufficient evidence of limb-specific responses consistent with a consummatory response.

Pain rating

All subjects were asked for their overall subjective rating of pain on their left and right side after each session in imaging study, using a scale ranging from 0 (no pain) to 10 (worst pain imaginable). The ratings were used to identify the existence of any consistent differences in pain experienced on either side, or habituation to stimuli in between sessions. The mean ratings for all sessions were: left 5.31±2.28, right 4.72±1.77 (paired t-test $p>0.2$). A two-way ANOVA was performed to test for differences in overall pain ratings among three sessions and/or lateralized pain. There were no significant effect of session number ($F(2,83) = 0.34$, $p = 0.71$), or left versus right pain ($F(2,83) = 1.41$, $p = 0.24$), or interaction between sessions and pain laterality ($F(2,83) = 0.04$, $p = 0.96$).

Computational model analysis

Identifying the processes underlying Pavlovian conditioning has been the subject of extensive study, and has aimed to determine what is actually being learned during conditioning, and how learning proceeds. This has established the importance of prediction errors and uncertainty, formalized by the classical learning theories of Rescorla-Wagner and Pearce-Hall, respectively [S18, 19]. Prediction errors are generated when outcomes deviate from expectations, and inform in what direction, and to what extent, future expectations should be changed so as to reduce future errors. In this way, they provide a teaching signal that continuously improves predictions based on experience. Uncertainty-based theories are based on the observation that learning is greatly enhanced when experience is limited and contingencies are novel. Uncertainty can be thought of as controlling learning rate - faster learning with greater uncertainty.

Computational neuroscience takes an explicitly mechanistic approach to the classic theories of experimental psychology, and hence aims to reverse engineer behavior to its constituent computational operations. Reinforcement learning models, such as temporal difference learning, describe ‘real-time’ algorithms that instantiate Rescorla-Wagner based learning rules into a ‘working’ learning system [S20]. Reinforcement learning models have had substantial success in describing the dynamic processes (both within and across trials) of Pavlovian learning: in eye-blink, reward, and aversive conditioning in animals and humans [S20, 21]. This extends both to the acquisition of behaviorally emitted conditioned responses, and also underlying neurophysiological activity. In humans, the application of computational models to human fMRI has allowed inferences about dynamic brain activity during learning, and has shown important translatable findings between humans and animal electrophysiology [S22].

In human fMRI studies of pain conditioning, the basic temporal difference model has been successfully applied to illustrate the existence of prediction error responses in the ventral putamen [S2]. More recently, Li et al [S5], and subsequently Boll et al [S23], showed that a modified temporal difference learning rule that also incorporates uncertainty (implemented as a learned ‘associability’ signal that controls the rate of learning) offers an account of both SCR acquisition and amygdala BOLD responses. This ‘hybrid’ model is attractive as it captures the key elements of both the original Rescorla-Wagner and Pearce-Hall learning theories, and functionally dissociates ventral striatum and amygdala activity (at least, in terms of BOLD responses). These studies also elegantly illustrate how physiological data (SCR) can be used to fit the parameters of the conditioning model, which is then subsequently applied to the brain data.

Here, we constructed Reinforcement learning models and used parameter fitting and model selection based on physiological data to identify the best model, and then used this to probe brain activity using trial-by-trial predictions of learning signals. This model can then be used to test competing hypotheses about the neural representation of preparatory (i.e. laterality non-specific) and consummatory (i.e. laterality specific) learning processes.

Standard Temporal Difference model

This model is the simple ‘real-time’ instantiation of the Rescorla-Wagner (RW) model. The value V of trial n+1 for a given cue j is updated based on the value of current trial n and the prediction error, difference between current value and outcome stimulus value R at trial n, weighted by a constant learning rate α [S24]:

$$V_j(n+1)=V_j(n)+\alpha\cdot(R(n)-V_j(n))$$

Here, the learning rate α is a free parameter within the range $0\leq\alpha\leq1$, determined by fitting to behavioral (SCR) data. The outcome value R on the current trial was determined by its valence, R(n)=1 (pain), or R(n)=0 (no pain). Assuming there is a preparatory learning system, which cannot distinguish lateralized outcomes, then R(n)=1 for all pain trials regardless of laterality. However, for a consummatory learning system, it is assumed that the outcomes were tracked separately for left and right, with the lateralized system tracking stimuli on its side only, ignoring the opposite side. For example, for the left system, R(n)=1 for left pain, or R(n)=0 for both right pain and no pain, and vice versa for the right system.

Hybrid temporal difference model

Proposed by Li et al. [S5], the hybrid model combines both Rescorla-Wagner and Pearce-Hall (PH) models, where the RW rule is used for error-driven value update and PH associability is used as a dynamic learning rate for RW to modulate predictive learning. Here, the PH associability is a changing quantity based on the average of the magnitude of recent prediction errors, and reflects an approximation of the effectiveness of a reinforcer based on its reliability in predicting reinforcement in the past. The value of associability decreases if the conditioned stimuli become correctly predictive of the stimuli outcome [S25]. The values of hybrid model were updated as follow:

$$V_j(n+1)=V_j(n)+\kappa\cdot\alpha_j(n)\cdot(R(n)-V_j(n))$$

$$\alpha_j(n+1)=\eta\cdot|R(n)-V_j(n)|+(1-\eta)\cdot\alpha_j(n)$$

Here, the learning rate directly equates to the associability α of given cue j at trial n+1, updated using the absolute value of prediction error from previous trial n and the associability at previous trial n. The free parameters α_0 (initial associability, $0\leq\alpha_0\leq1$), $\kappa(0\leq\kappa\leq1)$, $\eta(0\leq\eta\leq1)$, are determined by fitting to behavioral data. The prediction error between outcome value R and predicted value V was updated similarly as in the RW model, with the outcome valence determining outcome value R, which differs for the preparatory and consummatory learning system.

Model fitting and comparison

Previous studies have shown that SCRs can be sufficiently resolute to be used to fit trial-by-trial learning models (based on predictive value and/or associability) on a subject-by-subject basis [S5, 11, 23]. We followed the same approach here,

directly fitting the SCRs to the model value / associability, for parameter estimation and model comparison. Note that the same sequence of cues and outcomes in each session was presented to all subjects, to facilitate across-subject analysis.

Separately, for each individual session the free parameters of the models were optimized by estimating the maximum likelihood for individual subject's sequence of SCRs measured during that session. The likelihood of each trial's SCR was modeled as the normal distribution around a mean determined by the scaled predicted value (or associability, or the combination of both value and associability), computed by the model on that trial, plus a constant error term with an additional free parameter of distribution variance [S5]. To avoid contamination by pain over CS-predictive responses, only SCRs of no pain (i.e. unreinforced) trials were fitted with model outputs, but all trials were used in the computation of value and associability. Accordingly to our hypothesis (supported by the conventional SCR analysis), SCRs are preparatory conditioned responses, so in the model estimation, the pain outcome value is equivalent for left and right pain.

We adopted a hierarchical model fitting approach [S26]. Population free parameters were estimated from sets of fitted parameters from individual subjects. Initially we fit models using a separate set of free parameters for each individual subject. Suppose subject i comes from a population, whose free parameter set β_i is distributed following a population normal distribution $p(\beta_i|\mu_\beta,\sigma_\beta)$ with a mean of μ_β and variance of σ_β. By treating parameter fits from individual subjects as samples, the mean and variance of the population distribution can be estimated by fitting a normal distribution to these samples from their summary statistics. The optimal population free parameters can be obtained by iterating this process until sum of individual likelihood has reached maximum. Optimal population parameters were obtained for both TD and hybrid model, and the Bayesian information criterion (BIC) value was calculated for each model averaged across subjects and sessions, taking into account the number of free parameters available in each model with penalties, in order to quantitatively compare goodness of fit of these models. BIC was calculated using individual model parameters (Table S2).

The main objective of model fitting was to estimate population free parameters for subsequent imaging analysis. Due to the noisy nature of other behavioral data, only SCRs were chosen for fitting, and resulting free parameters were used for estimating outputs for consummatory learning systems as well. This incorporates an important assumption of our approach: i.e. that a potentially distinct consummatory response learning system will share the learning model and parameters (such as associability and learning rate) as the preparatory system. At least some justification of this assumption comes from the fact that consummatory eye-blink conditioning is well studied from the perspective of TD learning, and indeed these results originally formed the main body of evidence that motivated the application of Reinforcement Learning theory in animals [S20, 27]. Importantly, it has been shown elsewhere [S7] that the acquisition of limb EMG responses in shock conditioning follows a similar pattern to eye-blink responses (Note that Ref 6 used very large postural muscles in standing subjects, which allows much more refined recording and display of trial-by-trial responses).

Neuroimaging

A few specific points are noted here are about the precise application of the computational regressors. First, we chose to model the associability at the time of US presentation, since this should be the time at which learning is maximal as predictions are evaluated and updating proceeds. However, it is arguable that associability is first apparent at the time of cue presentation. Either way, the onset time makes little difference in reality because the ISI was short (1 second) in comparison to the BOLD response.

Second, it is important to note that the associability signal is updated by the outcome of the previous trial. This means that there is no correlation with the outcome on the current trial, so we can be confident that pain or movement on the current trial does not confound inferences about associability. The ISI EMG integral and its corresponding consummatory associability of the trial are not mutually correlated (mean correlation coefficient of EMG integral and associability series: ipsilateral $r(80)=0.02$, $p>0.4$, contralateral $r(80)=0.02$, $p>0.4$). This means that the cerebellar activity that we identify is not related to the motor response to pain or pain anticipation on that trial.

Third, in contrast, the prediction error is partially correlated with outcomes on the current trial, and is also a biphasic signal across the CS (cue) and US (outcome) occurring across a very short ISI (in temporal difference learning, the component of the prediction error at the time of the cue represents the increase in aversive value from moving from a baseline state in between trials, to the new predictive state when that trial's cue is first seen). In principle, this makes it difficult to reliably observe prediction error responses, and indeed this is the likely reason that we did not observe lateralized consummatory prediction errors in this study. It also makes it difficult to draw robust inferences about the precise nature of observed prediction error responses. Here, however, we draw on the fact that pain prediction errors are well known to exist in the ventral putamen from ours and other studies [S2, 3, 5], and so is a safe assumption. In particular, our study tests whether this is preparatory or consummatory, and distinguishing these two is unambiguous because they are largely anti-correlated (pain on the left side would cause a positive prediction error for a left consummatory system and negative prediction error for a right system, and vice-versa, Fig. S2).

Imaging acquisition and preprocessing

Functional MRI imaging data was acquired on a 3T Siemens Magnetom Trio scanner with Siemens standard 12 channel phased array head coil. Functional images were collected using a single-shot gradient echo EPI sequence (repetition time (TR) = 2500ms, echo time (TE) = 30ms, field of view = 240mm, flip angle = 80°). Thirty seven contiguous oblique-axial slices (3.75 × 3.75 × 3.75 mm voxels) parallel to the AC-PC line were acquired. Whole-brain high resolution T1-weighted structural images (256 × 256 × 208 with 1-mm isotropic voxels) were obtained.

Preprocessing and analysis of imaging data were conducted using SPM8 (Wellcome Trust Center for Neuroimaging, UK; http://www.fil.ion.ucl.ac.uk/spm/). Motion artefacts were removed by realigning images to the first scan of the first session using a 6 parameter rigid body transformation, and slice timing correction was performed. The mean functional image was then co-registered with the T1-weighted structural image, and subsequently normalized into MNI template space (SPM8 'segment' and 'normalize' estimated from structural images). After normalization images were re-sampled into 2×2×2 voxel sizes, followed by smoothing using a Gaussian kernel with full-width at half maximum (FWHM) of 8mm.

fMRI data analysis

The event related fMRI data were analyzed in a conventional computational model-based manner [S22]. We constructed sets of stick functions at the time of CS (visual cue) presentation and at the time of US (outcome - pain or pain omission) presentation for each trial in every session. We conducted a parametric analysis, in which the computational model was used to generate learning specific regressors as parametric modulators of these basic stick functions. As mentioned above, we used the best fitting model from the SCR-based analysis to generate these values, which was the hybrid model, with population free parameters for each session. This yields the following regressors:

Modelled at outcome time:
1) Preparatory associability $\alpha_{general}$
2) Left-sided consummatory associability α_{left}
3) Right-sided associability α_{right}
Modelled at cue and outcome time (this 'full' prediction error is a biphasic response)
4) Preparatory prediction error $VD_{general}$
5) Left-sided predicted error series VD_{left}
6) Right-sided prediction error series VD_{right}
Other regressors (of 'no interest')
7) Left pain delivery
8) Right pain delivery
9) Motion parameters (x6) derived from affine realignment in preprocessing

All of these regressors were compiled into one single GLM for first-level analysis for individual subject in SPM8. Results of these first level models were used in a second-level one-sample t-test to make a population inference in a standard manner (Figure 3). All fMRI statistics reported were from group random effects analysis.

Correction for multiple comparisons

We had specific hypothesis relating the function of the amygdala, ventral putamen and cerebellum. This comes from ours and other work that has specifically targeted computationally-modelled learning specific changes and reinforcement learning for pain (Table S1). Specifically, these were: bilateral amygdala [S28] (MNI x y z coordinates: 27 -5 -10, 18 -2 -16), ventral putamen [S2] (32 0 -8, -30 -2 -4), and cerebellum [S2] (28 -46 -30, -28 -46 -30). Small volume correction (SVC) for multiple comparison was conducted within anatomically defined 8mm diameter spherical masks built around these hypothesized structure coordinates. For display in the figures, statistical maps were presented at a threshold of $p<0.001$ uncorrected for multiple comparison, unless stated otherwise.

Cerebellum ROI analysis

Functional region of interest (ROI) analysis of the cerebellum was conducted using SUIT atlas (http://www.icn.ucl.ac.uk/motorcontrol/imaging/suit.htm, Spatially Unbiased Infratentorial Template [S29]). All T1-weighted structural scans were processed using SUIT toolbox in SPM8 to isolate the cerebellum and brain stem from the rest of the brain for each subject, producing masks of the cerebellum. Next, the masks were spatially normalized to the SUIT template. As for functional images, all first-level analyses were performed in space following standard realignment and slice time correction in SPM8. The resultant contrast images were then resliced into SUIT atlas space using previously generated SUIT normalization parameters. Spatial smoothing of the functional data was omitted in order to avoid activation from the visual cortex contaminating the cerebellum.

Second-level analysis was performed in SPM8 as previously described. SUIT statistical maps were presented at a threshold of p<0.001 uncorrected for multiple comparison, with an extent threshold of 5 contiguous voxels for partial correction, unless stated otherwise. The SUIT probabilistic MRI atlas of the human cerebellum was used to locate cerebellar lobules in analyses [S30].

In addition, post-hoc analyses of all ROIs were conducted by extracting beta estimates for each subject from the functional clusters of interest as they appeared on the statistical maps of a given model/contrast (corresponding peak coordinates were listed in Table S1), using MarsBaR toolbox (http://marsbar.sourceforge.net/). They were then averaged across subjects according to model or trial types without parametric modulation. To assess the validity of model values, trials were split into two categories (high and low), according to the model output values used as parametric modulator regressors (both associability and prediction errors). Contrasts were not parametrically modulated.

Supplemental References

S1. Bradley, M. M. (2009). Natural selective attention: Orienting and emotion. Psychophysiology *46*, 1–11.

S2. Seymour, B., O'Doherty, J. P., Dayan, P., Koltzenburg, M., Jones, A. K., Dolan, R. J., Friston, K. J., and Frackowiak, R. S. (2004). Temporal difference models describe higher-order learning in humans. Nature *429*, 664–667.

S3. Seymour, B., O'Doherty, J. P., Koltzenburg, M., Wiech, K., Frackowiak, R., Friston, K., and Dolan, R. (2005). Opponent appetitive-aversive neural processes underlie predictive learning of pain relief. Nat. Neurosci. *8*, 1234–1240.

S4. O'Doherty, J. P., Dayan, P., Friston, K., Critchley, H., and Dolan, R. J. (2003). Temporal Difference Models and Reward-Related Learning in the Human Brain. Neuron *38*, 329–337.

S5. Li, J., Schiller, D., Schoenbaum, G., Phelps, E. A., and Daw, N. D. (2011). Differential roles of human striatum and amygdala in associative learning. Nat. Neurosci. *14*, 1250–1252.

S6. Daum, I., Schugens, M. M., Ackermann, H., Lutzenberger, W., Dichgans, J., and Birbaumer, N. (1993). Classical conditioning after cerebellar lesions in humans. Behav. Neurosci. *107*, 748–756.

S7. Kaulich, T., Föhre, W., Kutz, D. F., Gerwig, M., Timmann, D., and Kolb, F. P. (2010). Differences in unconditioned and conditioned responses of the human withdrawal reflex during stance: Muscle responses and biomechanical data. Brain Res. *1326*, 81–95.

S8. Delgado, M. R., Li, J., Schiller, D., and Phelps, E. A. (2008). The role of the striatum in aversive learning and aversive prediction errors. Philos. Trans. R. Soc. B Biol. Sci. *363*, 3787–3800.

S9. Wolpert, D. M., and Ghahramani, Z. (2000). Computational principles of movement neuroscience. Nat. Neurosci. *3*, 1212–1217.

S10. McNally, G. P., Johansen, J. P., and Blair, H. T. (2011). Placing prediction into the fear circuit. Trends Neurosci. *34*, 283–292.

S11. Schiller, D., Levy, I., Niv, Y., LeDoux, J. E., and Phelps, E. A. (2008). From Fear to Safety and Back: Reversal of Fear in the Human Brain. J. Neurosci. *28*, 11517–11525.

S12. Criswell, E. (2011). Cram's Introduction to Surface Electromyography (Jones & Bartlett Learning).

S13. Littlewort, G. C., Bartlett, M. S., and Lee, K. (2009). Automatic coding of facial expressions displayed during posed and genuine pain. Image Vis. Comput. *27*, 1797–1803.

S14. Hamm, A. O., Greenwald, M. K., Bradley, M. M., and Lang, P. J. (1993). Emotional learning, hedonic change, and the startle probe. J. Abnorm. Psychol. *102*, 453–465.

S15. Ganesh, G., Franklin, D. W., Gassert, R., Imamizu, H., and Kawato, M. (2007). Accurate Real-Time Feedback of Surface EMG During fMRI. J. Neurophysiol. *97*, 912–920.

S16. Thieme, A., Thürling, M., Galuba, J., Burciu, R. G., Göricke, S., Beck, A., Aurich, V., Wondzinski, E., Siebler, M., Gerwig, M., et al. (2013). Storage of a naturally acquired conditioned response is impaired in patients with cerebellar degeneration. Brain, awt107.

S17. Timmann, D., Baier, P. C., Diener, H. C., and Kolb, F. P. (2000). Classically conditioned withdrawal reflex in cerebellar patients. 1. Impaired conditioned responses. Exp. Brain Res. *130*, 453–470.

S18. Mackintosh, N. J. (1983). Conditioning and associative learning (Clarendon Press Oxford).

S19. Bouton, M. E. (2007). Learning and behavior: A contemporary synthesis. (Sinauer Associates) Available at: http://psycnet.apa.org/psycinfo/2006-21409-000 [Accessed May 12, 2014].

S20. Sutton, R. S., and Barto, A. G. (1998). Introduction to Reinforcement Learning 1st ed. (Cambridge, MA, USA: MIT Press).

S21. Dayan, P., and Balleine, B. W. (2002). Reward, Motivation, and Reinforcement Learning. Neuron *36*, 285–298.

S22. O'doherty, J. P., Hampton, A., and Kim, H. (2007). Model-Based fMRI and Its Application to Reward Learning and Decision Making. Ann. N. Y. Acad. Sci. *1104*, 35–53.

S23. Boll, S., Gamer, M., Gluth, S., Finsterbusch, J., and Büchel, C. (2013). Separate amygdala subregions signal surprise and predictiveness during associative fear learning in humans. Eur. J. Neurosci. *37*, 758–767.

S24. Rescorla, R. A., and Wagner, A. R. (1972). A theory of Pavlovian conditioning: Variations in the effectiveness of reinforcement and nonreinforcement. Class. Cond. II Curr. Res. Theory, 64–99.

S25. Pearce, J. M., and Hall, G. (1980). A model for Pavlovian learning: variations in the effectiveness of conditioned but not of unconditioned stimuli. Psychol. Rev. *87*, 532.

S26. Daw, N. D. (2011). Trial-by-trial data analysis using computational models. Decis. Mak. Affect Learn. Atten. Perform. XXIII *23*, 3–38.

S27. Kehoe, E. J., and Joscelyne, A. (2005). emporally specific extinction of conditioned responses in the rabbit (Oryctolagus cuniculus) nictitating membrane preparation. Behav. Neurosci. *119*, 1011.

S28. Prevost, C., McNamee, D., Jessup, R. K., Bossaerts, P., and O'Doherty, J. P. (2013). Evidence for Model-based Computations in the Human Amygdala during Pavlovian Conditioning. PLoS Comput. Biol. *9*, e1002918.

S29. Diedrichsen, J. (2006). A spatially unbiased atlas template of the human cerebellum. Neuroimage *33*, 127–138.

S30. Diedrichsen, J., Balsters, J. H., Flavell, J., Cussans, E., and Ramnani, N. (2009). A probabilistic MR atlas of the human cerebellum. Neuroimage *46*, 39–46.
